# Strawberry seed extract and its major component, tiliroside, promote ceramide synthesis in the stratum corneum of human epidermal equivalents

**DOI:** 10.1371/journal.pone.0205061

**Published:** 2018-10-09

**Authors:** Shogo Takeda, Hiroshi Shimoda, Toru Takarada, Genji Imokawa

**Affiliations:** 1 Research & Development Division, Oryza Oil & Fat Chemical Co., Ltd., Aichi, Japan; 2 Research Institute for Biological Functions, Chubu University, Aichi, Japan; 3 Center for Bioscience Research and Education, Utsunomiya University, Tochigi, Japan; Kyungpook National University School of Medicine, REPUBLIC OF KOREA

## Abstract

The activation of peroxisomeproliferator-activated receptor (PPAR) α can stimulate the expression of ceramide-related enzymes, and a major component of strawberry seed extract (SSE) tiliroside enhances the expression of PPARα. We determined whether SSE and tiliroside may stimulate ceramide synthesis in the stratum corneum (SC) of the human epidermal equivalents (HEEs) culture model. Treatment with SSE at 1.0 and 3.0 μg/mL elicited a significant increase in the total ceramide content in the SC, which was accompanied by a significant increase in almost all ceramide species except for ceramide [EOS] and [AP]. Treatment with tiliroside at 0.3 μg/mL slightly accentuated the total ceramide content in the SC together with a significant increase in the ceramide [NS, NDS] content. Messenger RNA analysis demonstrated that SSE at 1 or 3 μg/mL significantly stimulated the gene expression of serine palmitoyltransferase (SPT) 2, ceramide synthase (CerS) 3, glucosylceramide synthase (GCS), and β-glucocerebrosidase (GBA) but not of SPT1, sphingomyelin synthase (SMS) 1/2 and acid sphingomyelinase (ASM). In contrast, tiliroside elicited significant increases in the gene expression levels of GCS and GBA only at 0.3 and/or 0.1 μg/mL. Western blotting analysis revealed that both SSE and tiliroside enhanced the protein expression levels of GCS and GBA but not of SPT2 at 1 or 3 and 0.1 or 0.3 μg/mL, respectively. These findings suggested that both SSE and tiliroside have a distinct potential to stimulate the level of ceramide [NS, NDS] in the SC by enhancing the expression of GCS and GBA. The higher stimulatory effect with SSE than tiliroside on SC ceramide synthesis correlates with the significant increase observed with SSE but not tiliroside in the gene expression levels of SPT2 and CerS3. Therefore, it is anticipated that SSE is effective in improving skin barrier function and moisture retention in several ceramide-deficit skin conditions, including surfactant-induced roughened skin, xerosis, and atopic dermatitis.

## Introduction

Ceramides are a type of sphingolipid and consist of a sphingoid base and a saturated fatty acid moiety. Ceramides are present as a dominant lipid in the stratum corneum (SC), the most upper layer of the epidermis of the skin and play a crucial role in its water-holding and barrier function. Up to now, more than 12 classes of ceramides have been assigned in human SC [1]. Non-genetic alteration in ceramide synthesis or metabolism leading to its deficiency or profile changes occurs in the epidermis of aged skin [2] and of patients with atopic dermatitis [3–11], which elicit xerosis and recurrent dermatitis, respectively, as a result of down-regulated function of ceramide in the SC. Thus, for amelioration of such dry skin conditions, it is important to compensate with orally or topically administered natural ceramide or by stimulating synthesis of ceramide in dry skin with xerosis and atopic dermatitis. Topical application of synthetic pseudo-ceramide has already been established as a means to efficiently ameliorate dry skin in xerosis and atopic dermatitis [12–19]. However, because of limitations in such topical application of ceramide in terms of its applied concentration and duration of efficacy, it could be more efficient to perform daily oral administration of food materials capable of stimulating ceramide synthesis, which results in an increase in ceramide mass in the SC.

Ceramides in the epidermis are synthesized by several enzymes such as serine palmitoyltransferase (SPT), ceramide synthase (CerS), glucosylceramide synthase (GCS), β-glucocerebrosidese (GBA), sphingomyelin synthase (SMS), and acid sphingomyelinase (ASM). SPT and CerS are involved in the *de novo* synthesis of ceramides. SPT catalyzes the condensation of serine and palmitoyl-CoA as the first step of *de novo* synthesis [20], and CerS catalyzes the formation of the basic ceramide structure by the *N*-acyltranslation of fatty acid [21]. Ceramides in the SC are synthesized from glucosylceramide and sphingomyelin by sequential enzymatic reactions of the sphingolipid metabolic enzymes, GCS, GBA, SMS, and ASM involved in synthesis or metabolism of these ceramide precursors. GCS plays a role in glucosylceramide synthesis [22] and glucosylceramide is hydrolyzed to ceramides by GBA [23,24]. SMS catalyzes the synthesis of sphingomyelin [25] and ASM yields ceramides by the hydrolysis of sphingomyelin [26]. Based upon the above sphingolipid metabolism in the epidermis, which is directly attributable to the production of ceramides in the SC, it is likely that stimulating the expression of these ceramide metabolic enzymes except for acid ceramidase is a key factor to increase ceramide level in the SC.

We have already developed a novel method, in which 3-dimensional human epidermal equivalents (3DHEEs) situated immediately before the development of the SC layers are used to evaluate the potential of candidate chemicals or materials to stimulate the expression of ceramide metabolic enzymes. This model results in the increased production of ceramide in the SC, which developed for 1 week after the initiation of the 3DHEEs culture [27]. Compared with the 3DHEEs accompanying the already developed SC, this model is likely to imitate *in vivo* keratinization processes and provides a precise evaluation of the stimulatory or inhibitory effects of chemicals or materials, including cytokines, on the quantity of ceramide in the SC [27]. Using this 3DHEEs model, as an implication for disrupted barrier mechanisms in atopic dermatitis, we have already demonstrated that Th1 cytokines accentuate but Th2 cytokines attenuate ceramide production in the SC of human epidermal equivalents [28].

Strawberry (*Fragaria ananassa*) seeds are generally ingested with its fruits pulp all over the world. However, published papers on the biological effects of strawberry seeds are limited in spite of the many studies on strawberry fruits. In a few cases, strawberry seed extract (SSE) has been reported to suppress oxidative stress [29] and to abrogate fat accumulation [29]. Previously, we found that in a constituent of strawberry seeds, tiliroside, is present as a major polyphenolic component (unpublished data). Tiliroside has been documented to exhibit anti-inflammatory [30–33], anti-hyperglycemic [34], hepatoprotective [35], and anti-diabetic activities [36]. Furthermore, tiliroside exhibits anti-obesity effects because of the enhancement of proliferator-activated receptor (PPAR) α expression [37]. In addition, the inhibitory effect of PPARα agonists and PPARγ ligands on cutaneous inflammation has been elucidated [38, 39]. The lineage connection between ceramide synthesis and PPAR activity in skin has been reported to indicate that PPARα, β/δ, and γ are involved in skin barrier function by stimulating keratinocyte differentiation and proliferation and epidermal lipid synthesis [40–42]. Furthermore, activation of PPARα has been demonstrated to increase ceramide content by accentuating the expression of SPT, GBA, SMS, and ASM [41, 43, 44]. Although the activation of PPARβ/δ up-regulates the expression of GBA to increase ceramide content, the activation PPARγ also stimulates the expression of GBA [41].

Based on the above evidence, it seems reasonable to assume that SSE and its major component, tiliroside, may stimulate ceramide synthesis in the cultured 3DHEEs via an activation of PPARα. Therefore, in this study, we have challenged this hypothesis. Here we show for the first time that SSE and tiliroside can differentially promote epidermal ceramide-metabolic enzymes, which results in the increased level of ceramide in the SC.

## Materials & methods

### Preparation of SSE and tiliroside

Dried strawberry seeds (*Fragaria ananassa* Duchesne var. Senga Sengana, Lot #51301) were purchased from GreenField Sp. z. o. o. Sp. k. (Warsaw, Poland). Prior to use, the strawberry seeds were processed as follows: they were separated from the fruit and pulp, then were washed, dried and packed (the age of the seeds was not determined). The chemical compounds in the strawberry seed extract (SSE) are shown in S1 Table. We obtained and confirmed the certification of analysis of the seeds that was provided by the vendor to authenticate the seeds. Additionally, we measured the polyphenol, lipid and tiliroside contents. One kg of the strawberry seeds was powdered and extracted with methanol (5 kg) at 80°C for 3 h. The extraction process was repeated twice, and the extracts were evaporated to yield the SSE (230 g, yield 23.0%). The content of tiliroside in the SSE was determined to be 10.2% (S1 Fig).

Tiliroside was isolated using the following procedure. SSE (230 g) was suspended in water (1 L) and extracted with ethyl acetate (500 mL) twice. Ethyl acetate solution was evaporated to obtain the ethyl acetate fraction (53.1 g, yield 5.3%). This ethyl acetate fraction (25.0 g) was fractionated by silicagel column chromatography (1.6 kg) with a mixture of chloroform and methanol (20:1 → 10:1) followed by mixture of chloroform, methanol and water (10:3:1 → 7:3:1), and then fraction 1 (18.7 g), 2 (2.2 g), 3 (1.2 g), 4 (0.8 g), 5 (2.3 g), 6 (0.6 g), and 7 (0.6 g) were obtained. Fraction 3 (200 mg) was purified by reversed-phase HPLC (COSMOSIL 5C18-MS-II, 20 ϕ × 250 mm; Nacalai Tesque, Kyoto, Japan) with mixture of acetonitrile and water (3:7) to obtain tiliroside (30 mg, yield 0.04%).

### Materials

The 3DHEEs culture model and assay medium were obtained from Japan Tissue Engineering Co., Ltd. (Aichi, Japan). 0.25 w/v% Trypsin-1 mmol/L/EDTA•4Na solution with phenol red (Trypsin/EDTA aqueous solution), phosphate-buffered saline (PBS) and skim milk were obtained from Wako Pure Chemical Co. Ltd. (Osaka, Japan). Ceramide standards of ceramide [NS, NDS] and [AS] were obtained from Matreya LLC. (State College, PA, USA). High-performance thin-layer chromatography (HPTLC) plates were purchased from Merck Millipore (Darmstadt, Germany). Fetal bovine serum (FBS) was purchased from Biosera (Boussens, France). NucleoSpin RNA II, PrimeScript Reverse Transcriptase and SYBR Premix Dimer Eraser were obtained from Takara Bio Inc. (Kusatsu, Japan). dNTP mixture and random primer were obtained from Invitrogen (Carlsbad, CA, USA). Radioimmunoprecipitation assay (RIPA) lysis buffer, protease phosphatase inhibitor cocktail, BCA protein assay kit, and Super Signal West Femto Maximum Sensitivity Substrate were purchased from Thermo Fisher Scientific Inc. (Waltham, MA, USA). Anti-serine palmitoyltransferase (SPT)2 antibody (Rabbit polyclonal, ab23696) and anti-glucosylceramide synthase (GCS) antibody (Rabbit polyclonal, H-300) were purchased from Abcam, Inc. (Cambridge, UK) and Santa Cruz Biotechnology, Inc. (Littleton, CO, USA), respectively. Anti-β-glucocerebrosidase (GBA) antibody (Rabbit polyclonal, G4171) and anti-β-actin antibody (Mouse monoclonal, A1978) were purchased from Sigma-Aldrich Co., LLC. (St. Louis, MO, USA). Horseradish peroxidase (HRP)-conjugated goat anti-rabbit IgG and HRP-conjugated goat anti-mouse IgG were purchased from Merck Millipore (Darmstadt, Germany).

### 3-Dimensional human epidermal equivalents cultured model

3DHEEs cultured model (LabCyte EPI-MODEL 6D) were cultured in pre-keratinization conditions before formation of the SC layer [45, 46]. Each cup of 3DHEEs models were placed into a 12- (for lipids analysis and western blotting) or 24-well (for real time qRT-PCR) plate, and assay medium was added to under the cup [47]. After incubating (37°C, 5% CO_2_ atmosphere) for 1 day, the medium was removed and fresh medium was added, and then SSE or tiliroside dissolved in 1% dimethyl sulfoxide was added to the medium. Only 1% dimethyl sulfoxide was added as a control. According to each experiment, the culture time was adjusted. Namely, the 3DHEEs models were cultured for 4, 5, or 7 days and subjected to real-time qRT-PCR, lipid analysis and western blotting, respectively. The medium was replaced every day.

### Lipid extraction

After 5 days' culture, the whole tissue was carefully peeled from the membrane. The removed tissue was incubated in trypsin/EDTA aqueous solution (37°C, 5% CO_2_ atmosphere) for 15 min to separate the SC from the epidermis. After adding FBS to inactivate trypsin activity, separation was carried out with the use of a microscope. The SC samples were washed with PBS and stored at -80°C until determinations were performed. Lipid extraction was carried out using a method described in previous reports [28, 48]. Namely, SC samples were homogenized using an ultrasonic homogenizer (AGC Techno Glass Co., Ltd. Haibara, Shizuoka) in a mixture of chloroform, methanol, and PBS (1:2:0.8) and mixed using a vortex mixer for 20 min. The mixtures were centrifuged (3000 rpm, 15 min) and then the supernatants were collected in test tubes. Chloroform (1 mL) and PBS (1 mL) were added to each supernatant and mixed for 20 min. After mixing, the mixture was centrifuged (3000 rpm, 15 min) and the bottom layer was collected using a glass syringe. The collected layer was dried at 30°C using N_2_ gas. The remaining precipitates were used for the quantification of total protein contents to correct for the ceramides contents.

### Thin layer chromatography

The ceramide content in the SC was measured by HPTLC. The previous method described by Sawada et al. [28] was modified and used for TLC analysis. The dried lipid samples were dissolved in a mixture of chloroform and methanol (2:1) and were developed on a TLC plate (10 × 10 cm). Lipid samples were developed twice. Namely, with a mixture of chloroform, methanol, and acetic acid (190:9:1) was used for the first development, and a mixture of chloroform, methanol, and acetic acid (197:2:1) was used for the second development. After development, the spots were visualized using 10% copper sulfate in 8% phosphoric acid aqueous solution, followed by heating at 180°C for 7 min. The spots for ceramides were scanned and analyzed using an imaging system (ImageQuant LAS500; GE Healthcare, Fairfield, CT, USA). The spot areas of ceramides were corrected for by the spot areas of ceramide standard.

### Real time qRT-PCR

The mRNA expression levels of SPT1, SPT2, CerS3, GCS, GBA, SMS1, SMS2, and ASM were measured by real-time qRT-PCR. Total RNA was extracted from the whole 3DHEEs model using NucleoSpin RNA II. After the extraction, 0.1 μg of total RNA was reverse-transcribed using PrimeScript Reverse Transcriptase to obtain cDNA. The real-time qRT-PCR reaction was conducted using SYBR Premix Dimer Eraser and Thermal Cycler Dice Real Time System Single (Takara Bio Inc., TM 800). The specific primers were used as follows; SPT1, 5′-GCGCGCTACTTGGAGAAAGA-3′ forward and 5′-TGTTCCACCGTGACCACAAC-3′ reverse; SPT2, 5′-AGCCGCCAAAGTCCTTGAG-3′ forward and 5′-CTTGTCCAGGTTTCCAATTTCC-3′ reverse; CerS3, 5′-CCAGGCTGAAGAAATTCCAG-3′ forward and 5′-AACGCAATTCCAGCAACAGT-3′ reverse; GCS, 5′-ATGTGTCATTGCCTGGCATG-3′ forward and 5′-CCAGGCGACTGCATAATCAAG-3′ reverse; GBA, 5′-TGGCATTGCTGTACATTGG-3′ forward and 5′-CGTTCTTCTGACTGGCAACC-3′ reverse; SMS1, 5′-CAACATTGGCGTAGACAT-3′ forward and 5′-TAGGAGGTACTCGTTCGTG-3′ reverse; SMS2, 5-ACTACTCTACCTGTGCCTGG-3 forward and 5-AGCAGCCAGCAGATTAAATG-3 reverse; ASM, 5′-TGGCTCTATGAAGCGATGG-3′ forward and 5′-AGGCCGATGTAGGTAGTTGC-3′ reverse; GAPDH, 5′-AAGGTGAAGGTCGGAGTCAAC-3′ forward and 5′-GGGGTCATTGATGGCAACAATA-3′ reverse. The mRNA expression level of each enzyme was corrected by expression level of GAPDH.

### Western blotting

Whole 3DHEEs models were homogenized in RIPA buffer containing protease phosphatase inhibitor cocktail and EDTA (0.5 M). The mixture was centrifuged (4°C, 13,000 rpm, 10 min), and the supernatant was collected. The protein contents of the supernatants were adjusted to 1.0 mg/mL and mixed with same volume of sample loading buffer (62.5 mM Tris-HCl, 5% 2-mercaptoethanol, 2% SDS, 25% glycerol and 0.01% bromophenol blue). After heating at 95°C for 5 min, the sample solution was electrophoresed on a 10% SDS-PAGE gel and then the separated protein was transferred to a polyvinylidene difluoride membrane. After blocking with 5% skim milk, the membrane was treated with a primary antibody followed by secondary antibody. Anti-SPT2 (1:1000), anti-GCS (1:200), anti-GBA (1:500), and anti-β-actin (1:10000) antibodies were used as primary antibodies. HRP-conjugated goat anti-rabbit IgG (1:10000) and HRP-conjugated goat anti-mouse IgG (1:10000) were used as secondary antibodies. Detection was performed using an imaging system (ImageQuant LAS500; GE Healthcare, Fairfield, CT, USA) and Super Signal West Femto Maximum Sensitivity Substrate.

### Protein analysis of the stratum corneum

Protein contents in SC in the 3DHEEs model were used for the correction of ceramide contents. Remaining precipitates obtained after lipid extraction were dissolved in a mixture of 10% SDS and 1 N NaOH (1:9) at 60°C for 2 h. Then mixture was neutralized with 2 N HCl, and the total protein amounts were determined using the BCA method.

### Statistics

All results are expressed as means ± standard error (SE). The significances of differences were examined by one-way analysis of variance (ANOVA) followed by Dunnett’s test. Value of *p* < 0.05 was considered statistically significant.

## Results

### Effect of SSE and tiliroside on ceramide contents in stratum corneum in 3DHEEs models

**Fig 1:**
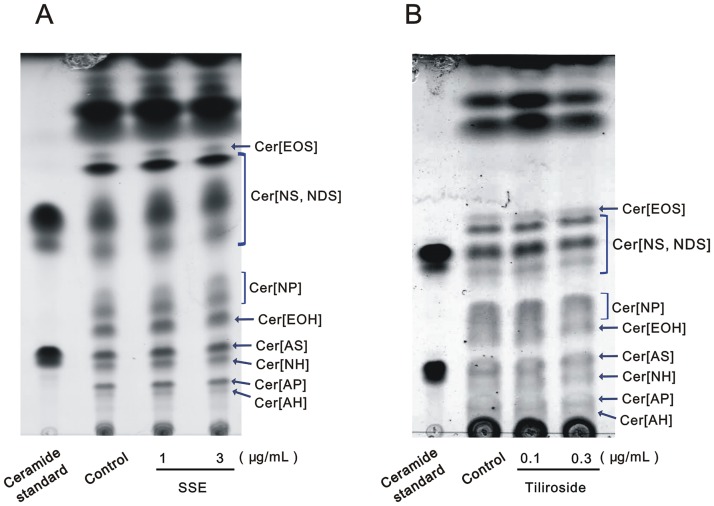
TLC analysis for lipids from the stratum corneum. TLC of SC lipids from 3DHEEs models treated with (A) SSE and (B) tiliroside. For measurement of SC ceramide (Cer), the lipid samples were developed twice on a TLC plate. Namely, a mixture of chloroform, methanol, and acetic acid (190:9:1) was used for the first development, and mixture of chloroform, methanol, and acetic acid (197:2:1) was used for the second development. The spots for ceramide were visualized using 10% copper sulfate in 8% phosphoric acid aqueous solution.

The ceramide composition in the SC of the 3DHEEs models treated with SSE or tiliroside were analyzed by HPTLC. HPTLC chromatograms (Fig 1) of the SC lipids after treatment with SSE or tiliroside demonstrated that ceramide species such as ceramide [EOS], [NS, NDS], [NP], [EOH], [AS], [NH], [AP], and [AH] were clearly detected and assigned in accordance with previous studies [28, 29].

**Fig 2:**
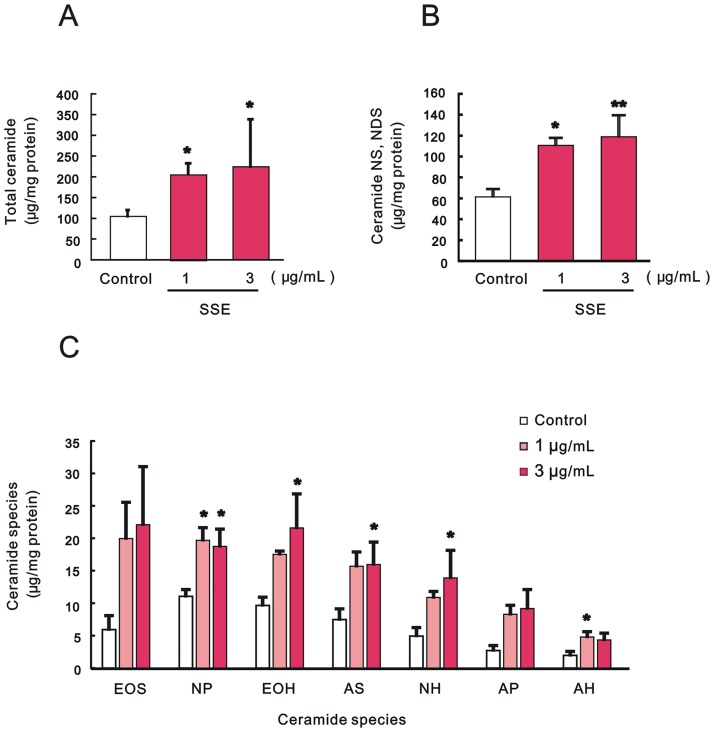
Effect of SSE on ceramide levels in SC. Effect on total ceramide (A), ceramide [NS, NDS] (B), and ceramide species except ceramide [NS, NDS] (C). 3DHEEs were treated with SSE (1 and 3 μg/mL) for 5 days, and the SC was separated from the epidermis and then total lipids were extracted. Ceramide contents were measured by TLC analysis and expressed as μg/mg protein. Each column represents mean and S.E. (n = 5). Significance of differences was examined by Dunnett’s test. Asterisks denote a significant difference from the control at * *p* < 0.05 and ** *p*< 0.01.

Quantitative analysis of ceramide revealed that treatment with SSE at concentrations of 1 and 3 μg/mL elicited a significant increase in the total ceramide content in the SC of the treated 3DHEEs (Fig 2A), which was accompanied by a significant increase in almost all the ceramide species (Fig 2B/2C) except for [EOS] and [AP].

**Fig 3:**
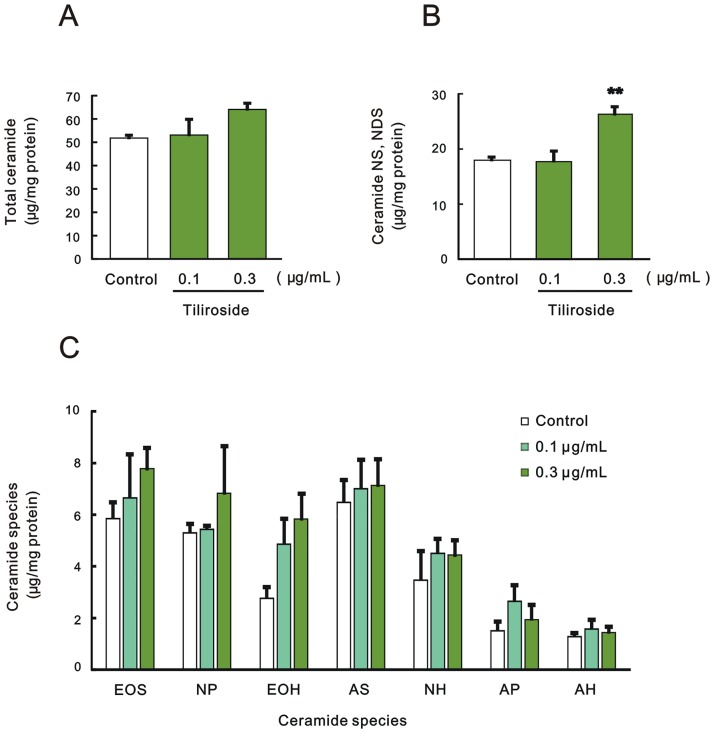
Effect of tiliroside on ceramide levels in the SC. Effect on (A) total ceramide, (B) ceramide [NS, NDS], (C) ceramide species except ceramide [NS, NDS]. 3DHEEs were treated with tiliroside (0.1 and 0.3 μg/mL) for 5 days, and the SC was separated from the epidermis and then total lipids were extracted. Ceramide contents were measured by TLC analysis and expressed as μg/mg protein. Each column represents mean and S.E. (n = 3–4). Significance of differences was examined by Dunnett’s test. Asterisks denote a significant difference from the control at **p < 0.01.

On the other hand, HPTLC analysis for tiliroside treatment (Fig 1B) revealed that tiliroside at a concentration of 0.3 μg/mL slightly but not significantly increased total ceramide contents (Fig 3A), accompanied by a significant increase in ceramide [NS, NDS] contents (Fig 3B). In terms of ceramide species except for ceramide [NS, NDS], tiliroside at concentrations of 0.1 and 0.3 μg/mL slightly but not significantly increased each ceramide species (Fig 3C).

### Effects of SSE and tiliroside on mRNA expression of ceramide synthesis-related enzymes involved in the de novo pathway

**Fig 4:**
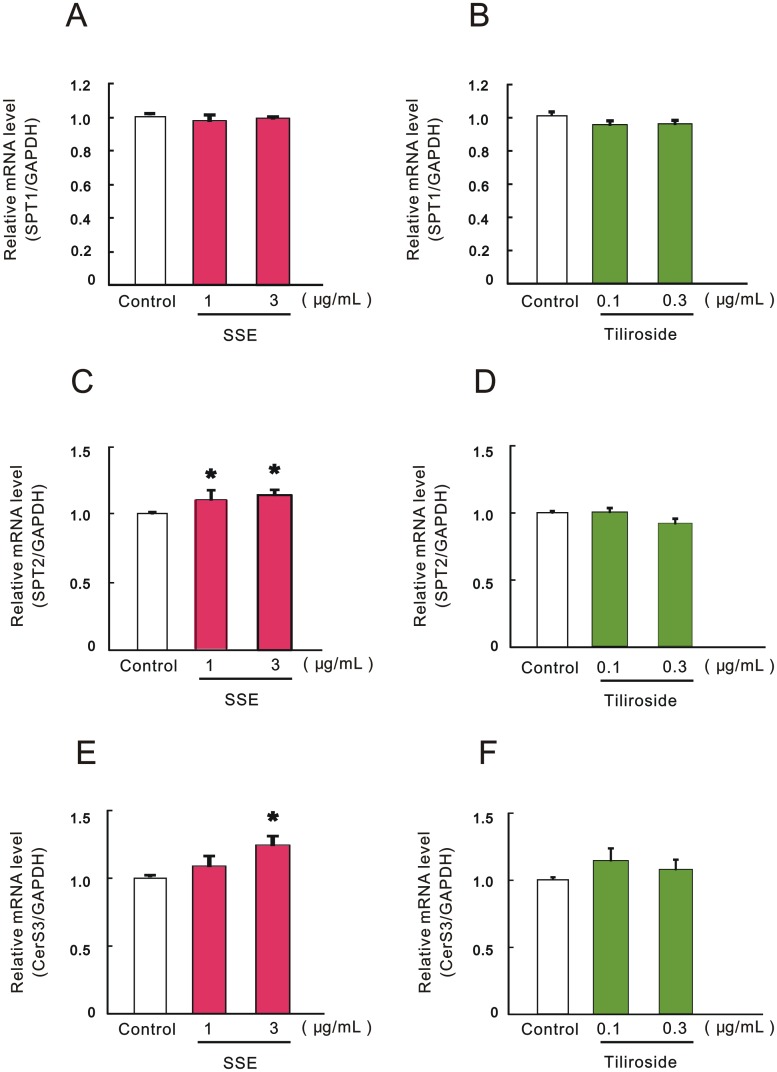
Effects of SSE and tiliroside on the mRNA expression levels of ceramide synthesis-related enzymes involved in the *de novo* pathway. 3DHEEs models were treated with SSE (1 and 3 μg/mL) or tiliroside (0.1 and 0.3 μg/mL) for 4 days, and total RNA was extracted. mRNA expression levels of SPT1 (A, B), SPT2 (C, D), and CerS3 (E, F) were determined by real-time qRT-PCR. The mRNA expression levels of GAPDH was used for correction of the each mRNA expression. Each column represents mean and S.E. (n = 3–4). Asterisks denote significant difference from control at *P < 0.05.

We next determined the effects of SSE and tiliroside on the mRNA level of ceramide synthesis-associated enzymes. Real-time qRT-PCR analysis revealed that SPT1 mRNA levels were not changed by treatment with SSE or tiliroside (Fig 4A/4B). On the other hand, the mRNA level of SPT2 was significantly increased by treatment with SSE at 1 and 3 μg/mL(Fig 4C), but not of tiliroside (Fig 4D). The mRNA level of CerS3, which is mainly involved in the synthesis of ceramide with long acyl chain moiety in the epidermis, was significantly up-regulated by treatment with SSE at a concentration of 3 μg/mL (Fig 4E). In contrast, tiliroside had no effect on mRNA expression of CerS3 at concentrations tested (0.1 and 0.3 μg/mL) (Fig 4F).

### Effect of SSE or tiliroside on the mRNA expression levels of GCS and GBA

**Fig 5:**
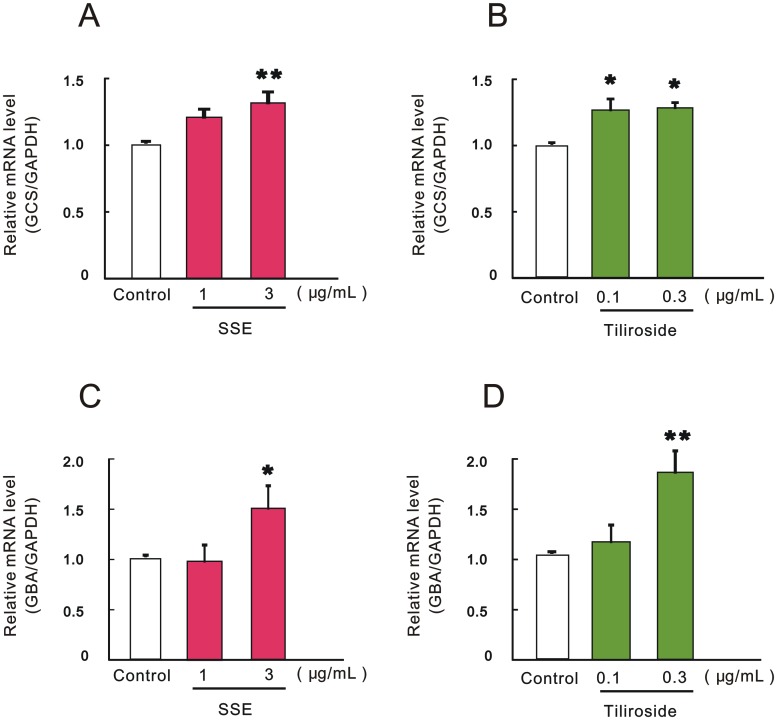
Effect of SSE and tiliroside on the mRNA expression levels of GCS and GBA. 3DHEEs models were treated with SSE (1 and 3 μg/mL) or tiliroside (0.1 and 0.3 μg/mL) for 4 days, and total RNA was extracted. mRNA expression levels of GCS (A, B) and GBA (C, D) were determined by real-time qRT-PCR. mRNA expression of GAPDH was used for correction of each mRNA expression. Each column represents mean and S.E. (n = 3–4). Asterisks denote significant difference from control at *P < 0.05 and **P < 0.01.

We next determined the effect of SSE or tiliroside on the mRNA expression levels of GCS and GBA. Real-time qRT-PCR revealed that each treatment with SSE at 3 μg/mL or with tiliroside at 0.1 and 0.3 μg/mL significantly increased the mRNA level of GCS (Fig 5A/5B). Similarly, each treatment with SSE at 3 μg/mL or with tiliroside at 0.3 μg/mL significantly increased the mRNA level of GBA (Fig 5C/5D).

### Effect of SSE and tiliroside on the mRNA expression levels of SMS and ASM

**Fig 6:**
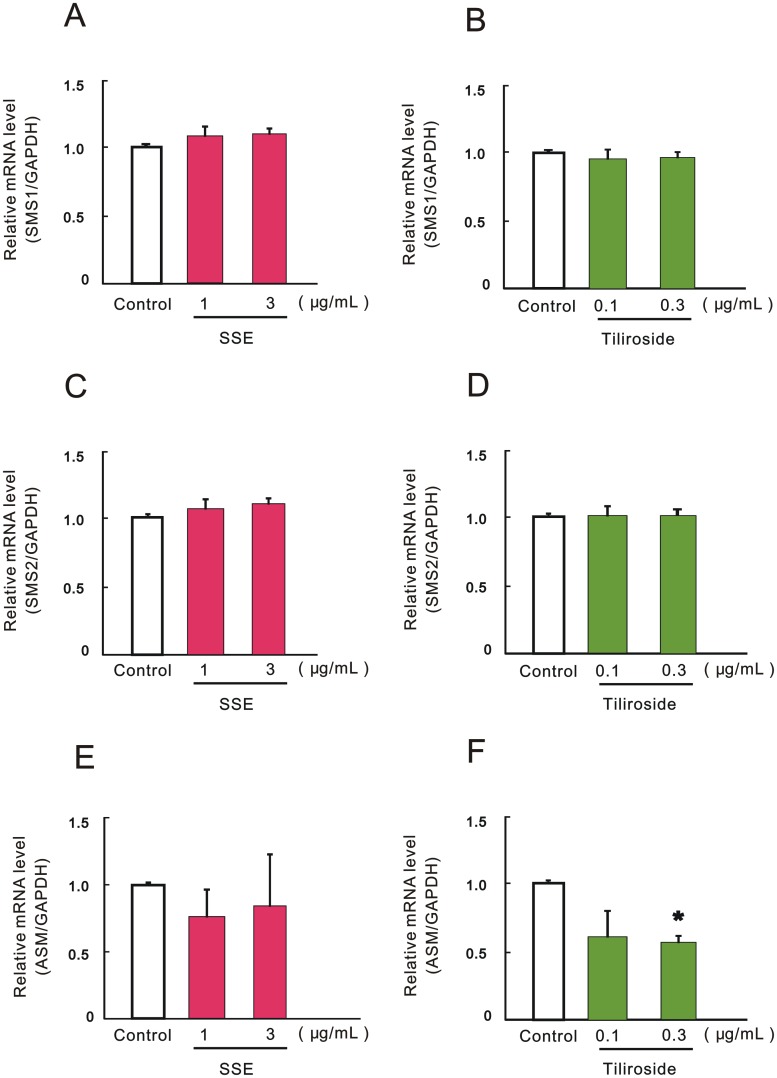
Effect of SSE and tiliroside on the mRNA expression levels of SMS and ASM. 3DHEEs models were treated with SSE (1 and 3 μg/mL) or tiliroside (0.1 and 0.3 μg/mL) for 4 days, and total RNA was extracted. The mRNA expression levels of SMS1 (A, B), SMS2 (C, D), and ASM (E, F) were determined by real-time qRT-PCR. mRNA expression of GAPDH was used for the correction of each mRNA expression. Each column represents mean and S.E. (n = 3, 4). Asterisks denote a significant difference from the control at *P < 0.05.

The mRNA expression levels of SMS1 were not changed by each treatment with SSE at 1 and 3 μg/mL or tiliroside at 0.1 and 0.3 μg/mL (Fig 6A/6B). Similarly, each treatment with SSE at 1 and 3 μg/mL or tiliroside at 0.1 and 0.3 μg/mL had no effect on the mRNA level of SMS2 (Fig 6C/6D). On the other hand, the mRNA level of ASM was slightly decreased by treatment with SSE at 1 and 3 μg/mL (Fig 6E), but significantly suppressed by treatment with tiliroside at 0.3 μg/mL (Fig 6F).

### Effects of SSE and tiliroside on the protein expression levels of SPT2, GCS, and GBA

**Fig 7:**
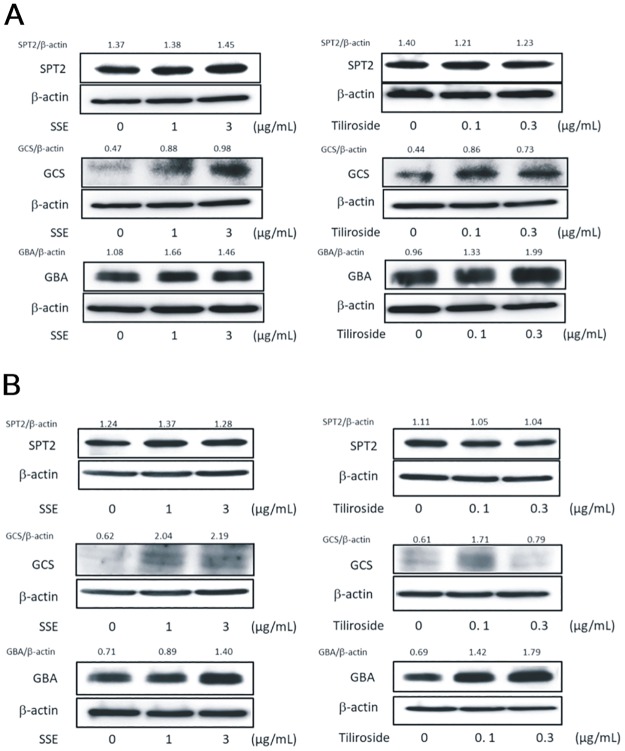
Effect of SSE and tiliroside on the protein expression levels of SPT2, GCS, and GBA. 3DHEEs models were treated with SSE (1 and 3 μg/mL) or tiliroside (0.1 and 0.3 μg/mL) for 5 (A) and 7 (B) days and total protein was extracted. The protein expression levels of SPT2, GCS and GBA were determined by western blotting.

We next determined the effects on the protein expression level of these ceramide synthesis-related enzymes. While the protein expression levels of GCS and GBA at day 5 of culture were distinctly increased by the treatment of SSE at 1 and 3 μg/mL(Fig 7A and S2 Fig), those at day 7 of culture were also up-regulated at 1 and 3 μg/mL and 3 μg/mL, respectively (Fig 7B and S3 Fig). On the other hand, the protein expression levels of SPT2 at both 5 and 7 days of culture were not changed by with treatment SSE at 1 and 3 μg/mL (Fig 7A/7B). Whereas tiliroside had no effect on the protein expression level of SPT2, the protein expression levels of GCS and GBA at both 5 and 7 days of culture were distinctly increased by treatment with tiliroside at 0.1 and/or 0.3 μg/mL (Fig 7A/7B). The protein expression of CerS3 was at undetectable level at both 5 and 7 days of culture (S4 Fig).

## Discussion

**Fig 8:**
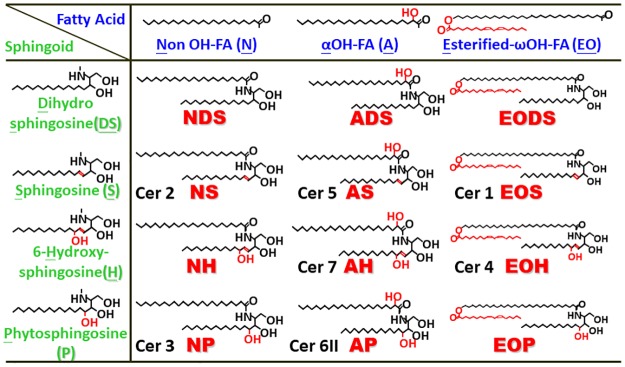
Chemical structure of ceramide species. In this study, we evaluated the effect of SSE and its major component, tiliroside, on ceramide content in the SC and the expression of ceramide synthesis-related enzymes in a new reconstructed human epidermal keratinization model (3DHEEs), which we developed to analyze factors that regulate ceramide synthesis, especially in the SC of human skin [27]. We found that treatment with SSE at concentrations of 1 and 3 μg/mL significantly increased the total ceramide content in the treated 3DHEEs in concert with significantly increased level of ceramide species, such as ceramide [NS/NDS], [NP], [NH], [EOH], [AS], and [AH]. Similarly, tiliroside at a concentration of 0.3 μg/mL slightly increased total ceramide contents with a significantly increased level of ceramide [NS, NDS]. These results indicated that the both SSE and tiliroside increase the ceramide [NS, NDS] content in the SC, which is assigned as the largest component among ceramide species [27]. Whereas it is likely that tiliroside is an active component in the SSE that enhances ceramide [NS/NDS] production, it remains uncertain whether tiliroside and/or other unknown compounds are active components that enhance the production of other ceramide species. Therefore, further study is required to determine the active components that increase the content of other ceramide species. Because ceramide [NS, NDS] analogs elicit preferential improvements in the skin barrier function, including moisture retention [50], it is anticipated that the topical or oral administration of SSE and tiliroside may enhance the moisture retention and barrier function of skin by up-regulating ceramide (especially ceramide [NS, NDS]) production in the SC.

Ceramides in human SC can be divided into 11 groups according to their fatty acid and sphingoid base structures [1], as depicted in Fig 8: ceramide [NDS] contains non-OH fatty acids [N] and dihydrosphingosines [DS]; ceramide [NS] contains [N] and sphingosines [S]; ceramide [NH] contains [N] and 6-hydroxy sphingosines [H]; ceramide [NP] contains [N] and phytosphingosines [P]; ceramide [ADS] contains α-OH fatty acids [A] and [DS]; ceramide [AS] contains [A] and [S]; ceramide [AH] contains [A] and [H]; ceramide [AP] contains [A] and [P]; ceramide [EOS] contains ester-linked fatty acids, and ω-OH fatty acids [EO] and [S]; ceramide [EOH] contains [EO] and [H]; and ceramide [EOP] contains [EO] and [P]. These classes can be further subdivided into many species based on their chain length. To date, 350 species of ceramides have been identified in human SC using normal-phase liquid chromatography coupled with electrospray ionization–mass spectrometry [1, 49]. Although the role of each ceramide species has not been clearly studied, acylceramides such as ceramide [EOS] and [EOH] is well known to be more distinctly associated with barrier function in SC than the other ceramides [2].

**Fig 9:**
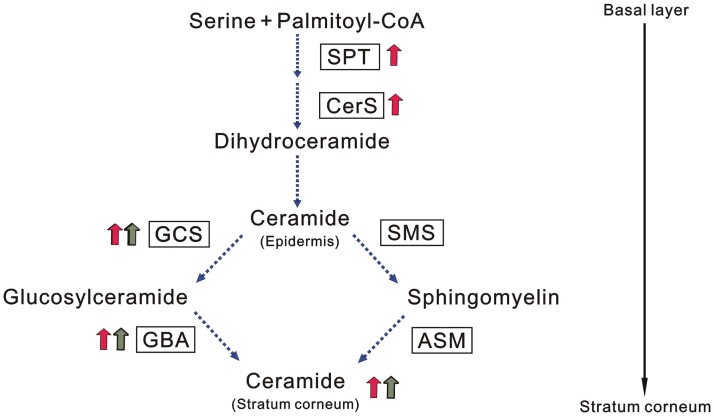
SC ceramide synthesis-acceleration mechanism of SSE and tiliroside. Increased expression by SSE (Red arrows) and tiliroside (Green arrows). SSE increased expression of SPT, CerS, GCS, and GBA. Tiliroside increased expression of GCS and GBA.

SC ceramides are synthesized through two different pathways which occur via glucosylceramide or sphingomyelin as intermediate products required for further hydrolysis by GBA and ASM, respectively, to produce ceramides [22]. Thus, as depicted in Fig 9, intracellular ceramide synthesis is initiated by the action of SPT1/2 to produce sphinganine, which is converted to intracellular ceramides as an intermediate precursor required for the formation of sphingomyelin and glucosylceramide by CerS, in which CerS3 acts as a predominant isozyme in epidermal keratinocytes. After intracellular ceramide synthesis by CerS, SMS triggers the synthesized ceramides to produce sphingomyelin, which is in turn hydrolyzed by ASM at the interface between granular cells and SC cells to yield ceramides in the SC [51]. On the other hand, GCS transfers glucose to ceramides or acylceramides to produce glycosylceramide or glucosylacylceramide, which is hydrolyzed by GBA at the interface between granular cells and SC cells to yield ceramides or acylceramides in the SC. The glucosylceramide pathway occurs as a major part (at least two-thirds) of SC ceramide synthesis [22, 51] and acylceramides such as ceramide [EOS, EOH] are synthesized from acylglucosylceramide, to which is converted only in the glucosylceramide pathway by the action of GBA [51].

As for the biological mechanisms involved in up-regulated level of ceramide [NS, NDS] by SSE and tiliroside, we next characterized the effect of SSE and tiliroside on the expression of enzymes involved in ceramide synthesis. While the mRNA expression levels of SPT2, CerS3, GCS, and GBA among the metabolic enzymes were significantly up-regulated by SSE, GCS and GBA only were accentuated at the transcriptional levels by tiliroside. Consistent with their increased gene expression, the protein expression levels of GCS and GBA were also up-regulated by treatment with both SSE and tiliroside. These results suggest that both SSE and tiliroside elicit an increase in the level of ceramide [NS, NDS] in the SC by enhancing the expression of at least GCS and GBA, which play a predominant role in SC ceramide synthesis via the glucosylceramide pathway. Furthermore, the slight and significant increases in acylceramides such as ceramide [EOS] and [EOH], respectively, in the SC of SSE-treated 3DHEEs strongly support the possibility that SSE predominantly stimulates the glucosylceramide pathway to elicit increases in acylceramides in the SC. It is likely that the higher stimulatory effect by SSE than tiliroside on SC ceramide synthesis is in a good agreement with the observation that the gene expression levels of SPT2 and CerS3 accentuated by treatment with SSE, but not with tiliroside, because these enzymes are involved in both pathways mediated via glucosylceramide or sphingomyelin as intermediate products, leading to ceramide synthesis in the SC. It is likely that the significant suppressive effect observed by tiliroside but not by the SSE on the mRNA expression level of ASM is also associated with the weaker stimulatory effect of tiliroside on SC ceramide synthesis than the effect of SSE.

In the lineage connection between epidermal lipid synthesis including ceramide synthesis and PPAR activity, PPARα activators stimulate lipid synthesis in human epidermal organotypic cultures [44]. Activators of PPARα, β/δ, and γ, and LXR accelerate permeability barrier recovery after acute barrier disruption [40–42, 52, 53]. The accelerating effect on barrier recovery is consistent with the evidence that the activation of PPARα, β/δ, and γ also induces the enhancement of epidermal lipid synthesis, including ceramide synthesis, lammellar granule (LB) secretion, and the activity of GBA, an enzyme required for the extracellular processing of secreted LB contents to yield SC ceramides [40]. Although PPARγ activators fail to increase ceramide synthesis, the synthesis of all three classes of lipids required for LB formation, cholesterol, free fatty acids, and ceramides, is distinctly accentuated by treatment with PPARα, PPARβ/δ, and LXR activators [40–42, 52, 53]. Because tiliroside was reported to enhance PPARα expression [36], it seems reasonable to assume that the increased gene and protein expression of GBA in SEE- and tiliroside-treated 3DHEEs is attributable in a major part to the possible activation of PPARα by SSE and tiliroside. Because SSE used in this study contains tiliroside by approximately 10%, it is likely that the significant increase in the gene expression levels of SPT2 and CerS3, which was provoked by SSE but not by tiliroside, may be ascribed to possible activation of PPAR by unknown components other than tiliroside, although the questions as to what PPARs are associated with the increased expression of SPT or Cer3 by SSE and whether PPARα substantially plays an essential role in up-regulating the expression of GCS and GBA are beyond the scope of the present study.

In conclusion, our results demonstrated that treatment with SSE and its major component, tiliroside, distinctly increased the ceramide content in the SC by enhancing the expressions of ceramide synthesis-related enzymes required for the glucosylceramide pathway, such as GCS and GBA. Furthermore, SSE elicited greater increases in the SC ceramide content than tiliroside by additionally stimulating the expression of SPT2 and CerS3, common enzymes required for the formation of both glucosylceramide and sphingomyelin as an intermediate precursor of SC ceramide synthesis. Therefore, it is anticipated that SSE may be effective in improving skin barrier function and moisture retention in several ceramide-deficit skin conditions including surfactant-induced roughened skin, xerosis, and atopic dermatitis.

## Supporting information

S1 Fig TLC and HPLC chromatogram of SSE. (DOCX)

S2 Fig Supporting data for Fig 7A. 3DHEEs models were treated with (A) SSE (1 and 3 μg/mL) or (B) tiliroside (0.1 and 0.3 μg/mL) for 5 days and total protein was extracted. The protein expression levels of SPT2, GCS and GBA were determined by western blotting. Data represent the original uncropped and unadjusted blots in the Fig 7A.
(TIF)

S3 Fig Supporting data for Fig 7B. 3DHEEs models were treated with (A) SSE (1 and 3 μg/mL) or (B) tiliroside (0.1 and 0.3 μg/mL) for 7 days and total protein was extracted. The protein expression levels of SPT2, GCS and GBA were determined by western blotting. Data represent the original uncropped and unadjusted blots in the Fig 7B.
(TIF)

S4 Fig Supporting data for data not shown Fig. (TIF)

S1 Table The contents of chemical compounds and nutrition facts in SSE. (TIF)

## Abbreviations

3DHEEs: 3-dimensional human epidermal equivalents
ASM: acid sphingomyelinase
CerS: ceramide synthase
GBA: β -glucocerebrosidase
GCS: glucosylceramide synthase
PPAR: peroxisomeproliferator-activated receptor
SC: stratum corneum
SMS: sphingomyelin synthase
SPT: serine palmitoyltransferase
