# Supplementary material for: Strawberry seed extract and its major component, tiliroside, promote ceramide synthesis in the stratum corneum of human epidermal equivalents
Source: PLoS One. 2018 Oct 9;13(10):e0205061. doi: 10.1371/journal.pone.0205061 (PMC6177135; doi:10.1371/journal.pone.0205061)
Supplement: S1 Fig — (DOCX) [file pone.0205061.s001.docx]

**Supporting information**


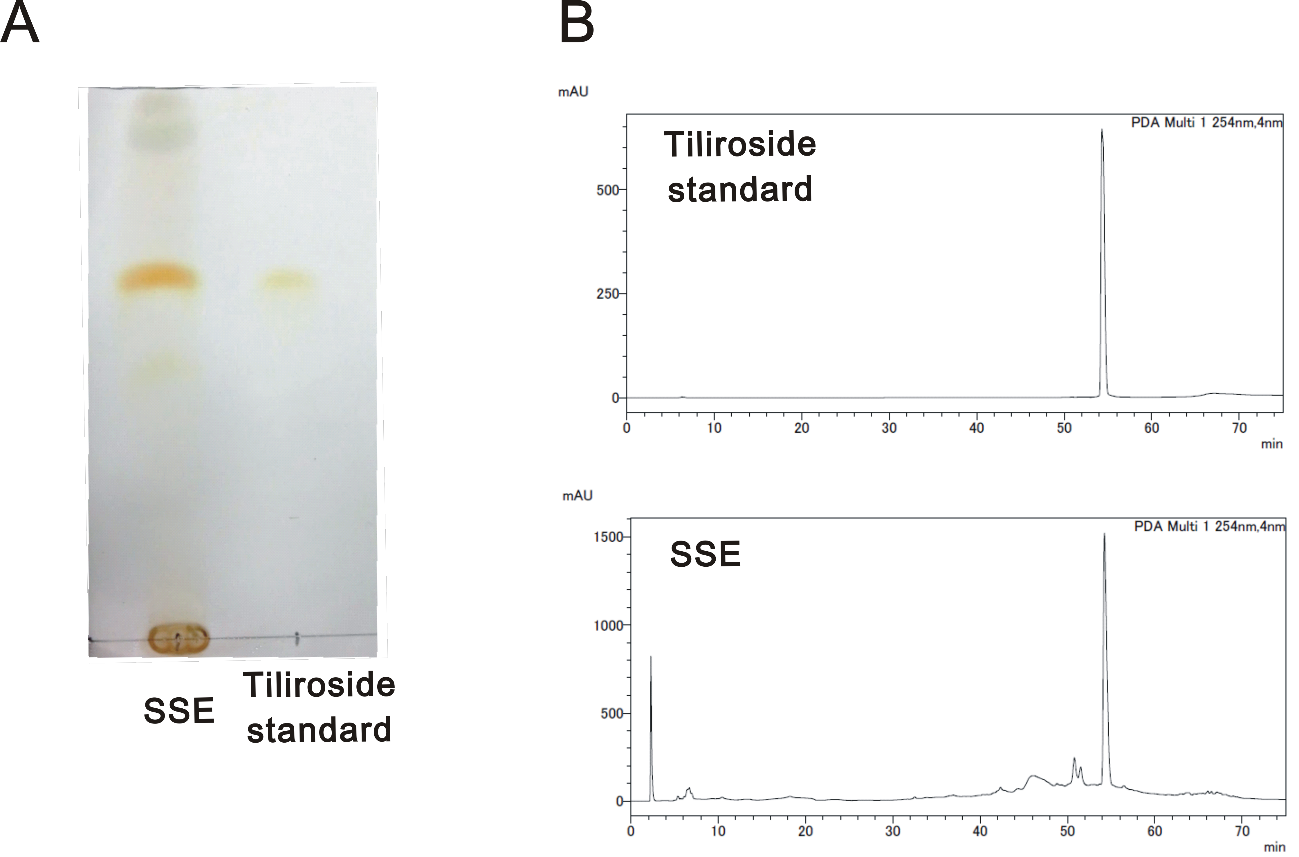


**S1 Fig. TLC and HPLC chromatograms of SSE**

A) TLC chromatograms of SSE (left lane) and tiliroside standard (right lane). Samples were developed by mixture of chloroform and methanol (8:2). Spot for tiliroside was visualized by 10% phosphoric acid aqueous solution followed by heating (180°C). B) Reversed HPLC chromatogram of SSE (upper) and tiliroside standard (bottom). CAPCELL PAK C18 SG-120 (4.6ϕ×250 mm; Osaka Soda Co., Ltd., Osaka, Japan) was used for analysis. Water and methanol were used for solvent A and B, respectively. Gradient condition was 0 to 60 min (solvent B: 5-100%). Column temperature was set at 35℃. Flow late was fixed at 1.0 mL/min. An UV detector (254 nm) was used.
