# Supplementary figures and images for: Strawberry seed extract and its major component, tiliroside, promote ceramide synthesis in the stratum corneum of human epidermal equivalents
Source: PLoS One. 2018 Oct 9;13(10):e0205061. doi: 10.1371/journal.pone.0205061 (PMC6177135; doi:10.1371/journal.pone.0205061)

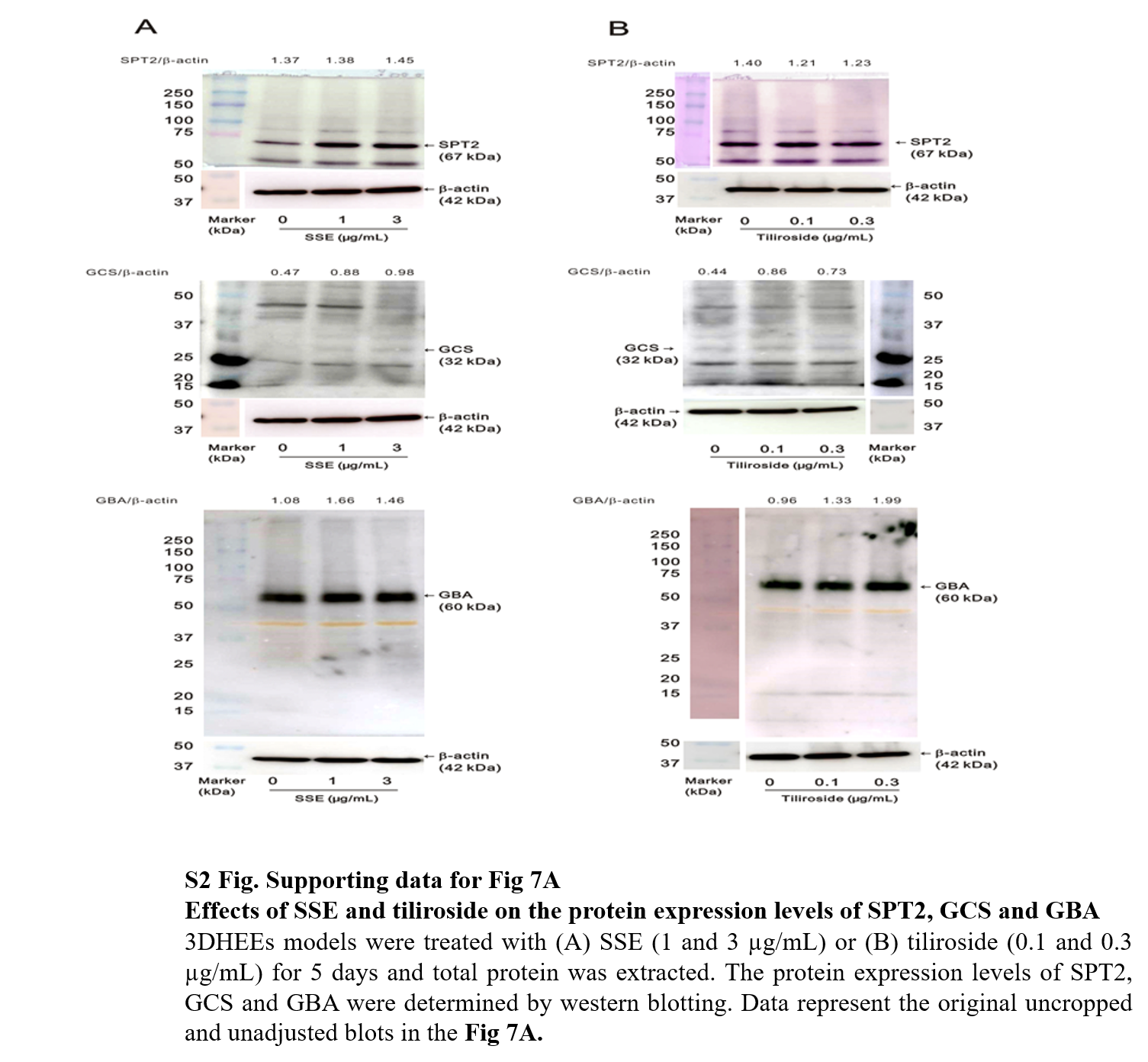

Supplement: S2 Fig — 3DHEEs models were treated with (A) SSE (1 and 3 μg/mL) or (B) tiliroside (0.1 and 0.3 μg/mL) for 5 days and total protein was extracted. The protein expression levels of SPT2, GCS and GBA were determined by western blotting. Data represent the original uncropped and unadjusted blots in the Fig 7A. (TIF) [file pone.0205061.s002.tif]

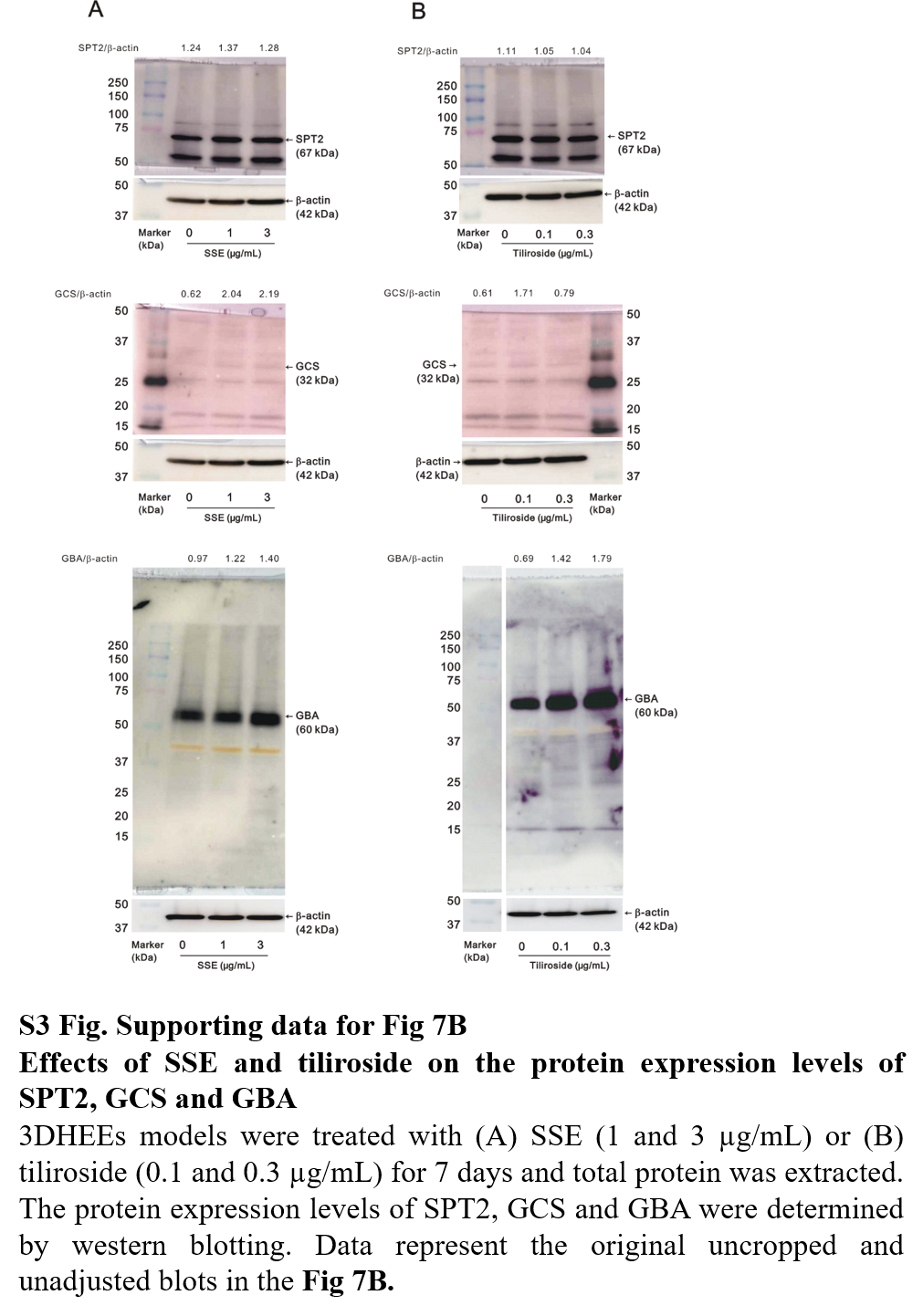

Supplement: S3 Fig — 3DHEEs models were treated with (A) SSE (1 and 3 μg/mL) or (B) tiliroside (0.1 and 0.3 μg/mL) for 7 days and total protein was extracted. The protein expression levels of SPT2, GCS and GBA were determined by western blotting. Data represent the original uncropped and unadjusted blots in the Fig 7B. (TIF) [file pone.0205061.s003.tif]

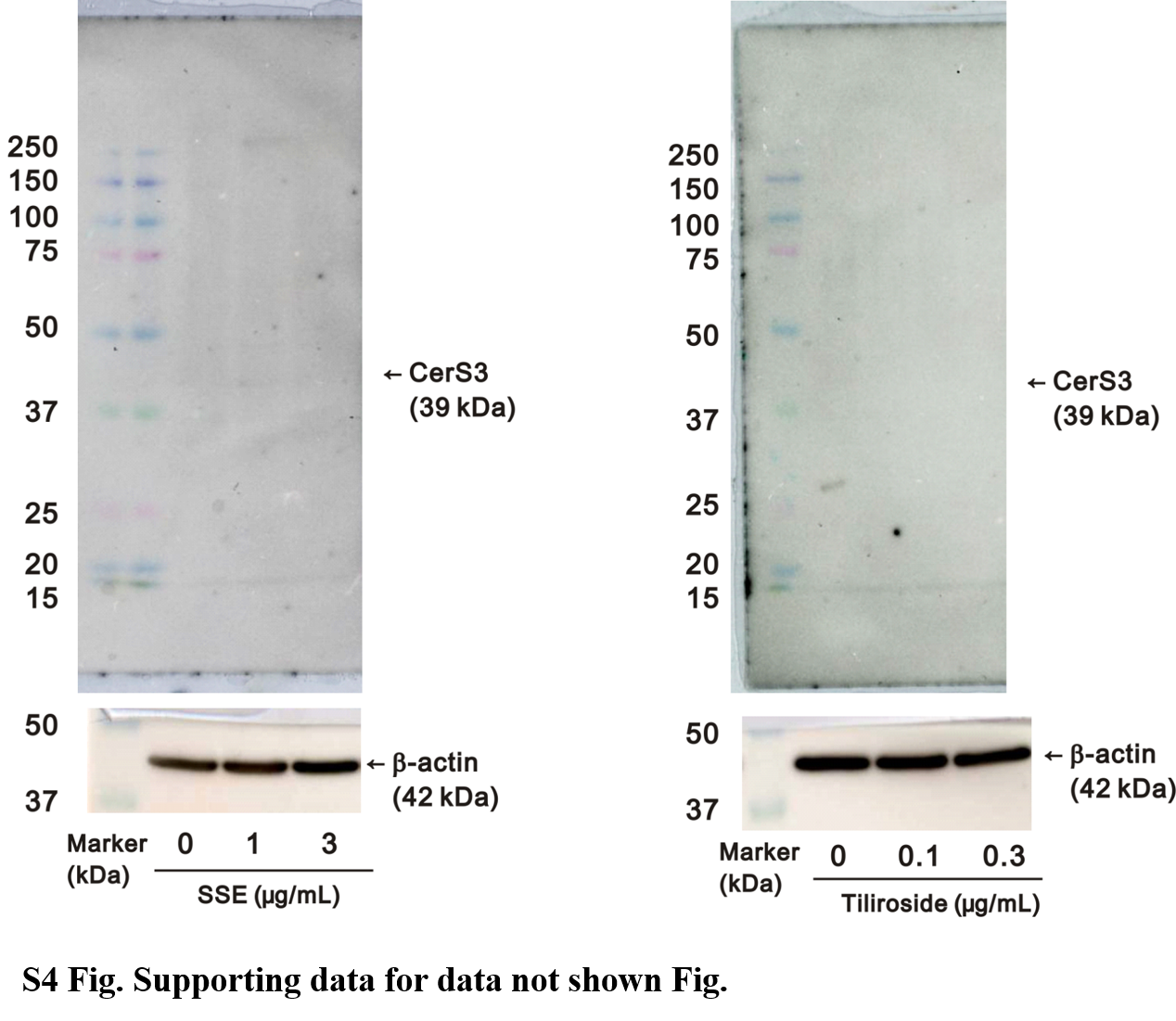

Supplement: S4 Fig — (TIF) [file pone.0205061.s004.tif]

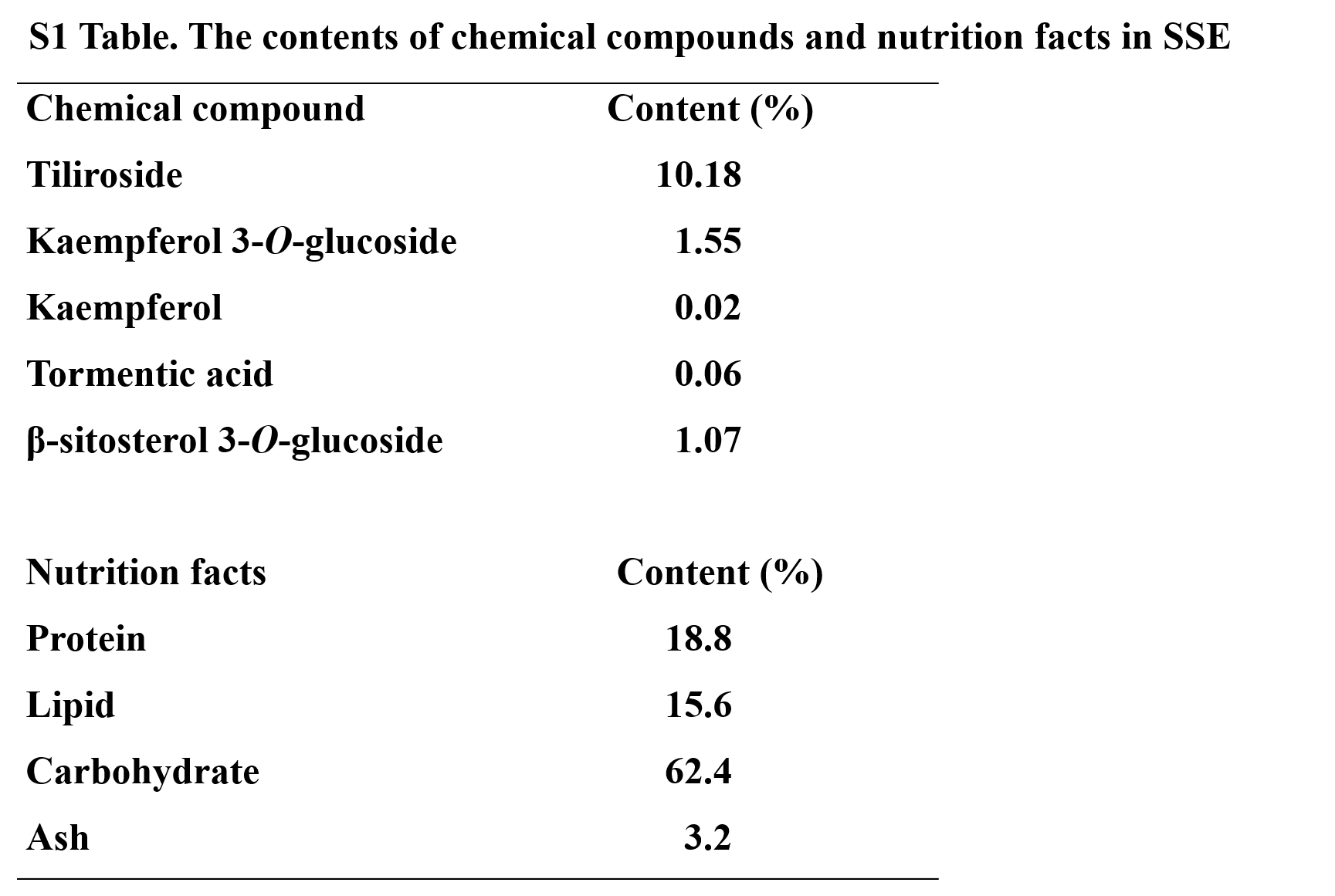

Supplement: S1 Table — (TIF) [file pone.0205061.s005.tif]
